# Perspective on the health value of carbohydrate-rich foods: glycemic index and load; fiber and whole grains

**DOI:** 10.1016/j.ajcnut.2024.07.004

**Published:** 2024-09-02

**Authors:** David JA Jenkins, Walter C Willett

**Affiliations:** 1 Department of Nutritional Sciences and Medicine, Temerty Faculty of Medicine, University of Toronto, Toronto, Ontario, Canada; 2 Li Ka Shing Knowledge Institute, St. Michael’s Hospital, Toronto, Ontario, Canada; 3 Department of Epidemiology, Harvard T.H. Chan School of Public Health, Boston, MA, United States; 4 Department of Nutrition, Harvard T.H. Chan School of Public Health, Boston, MA, United States

**Keywords:** quality of carbohydrate-rich foods, plant foods, dietary recommendations, diabetes, cardiovascular disease

## Abstract

**Background:**

For over 45 y increasingly comprehensive food tables of glycemic index (GI) and glycemic load (GL) have been published in the *American Journal of Clinical Nutrition* to determine the GI and GL values of diets. Recently the WHO based on a 2019 series of meta-analyses concluded that increases in dietary fiber and whole grains but not reduction in GI or GL warranted recommendations for chronic disease reduction.

**Methods and results:**

We therefore provide a perspective on the current evidence that indicates that GI and GL are also determinants of risk of chronic disease outcomes. We are also concerned with the term dietary fiber used in the singular when there are many dietary fibers that may differ in their physiological effects. Furthermore, the term “whole grains” that refers to “whole grain flour” limits the exploration of “intact” grains that are low GI and have useful physiological effects.

**Conclusion:**

We conclude that all these determinants of the health values of carbohydrate-rich foods should be used in combination to assess the health value of carbohydrate-rich foods.

It is now over 45 y since the concept of the glycemic index (GI) was first published in the *American Journal of Clinical Nutrition* with a limited GI table that provided the GI classification for 63 common foods [1]. The stimulus, for the creation of the GI, was the increasing popularity of “low carb diets”: Atkins, Zone, Paleo, and Keto diets. Because carbohydrates came from plant foods, it was reasoned that unless a health value could be ascribed to carbohydrate-rich foods, the movement to more plant-based diets would be more difficult to justify because carbohydrate consumption, which represents 50% of the world’s energy intake internationally, would have limited appeal.

Following the initial GI table and much further research, greatly expanded international GI tables have been published, again in the *American Journal of Clinical Nutrition*, with over 4000 foods, including dishes from many different cultures that can be used to document the GI values of diets eaten around the world [2]. These data have allowed the GI classification of diets from food frequency questionnaires and the assessment of disease associations in large prospective cohort studies.

Recently, relationships between GI and the associated glycemic load (GL) (GI × weight of the dietary carbohydrates in the food or diet/100) [3,4] with disease outcomes have been challenged by the WHO [5]. It was concluded that the evidence for disease associations was robust for fiber and whole grains, but insufficient for GI and GL such that no recommendation was possible. Interestingly, this conclusion was based on a WHO-sponsored, systematic review and meta-analyses that indicated significant findings in the appendix table that were not reflected in the conclusions of the article [6]. In Supplementary Table D:1 (page 51) [6], significant GI associations were reported for cardiovascular disease (CVD) mortality [RR 1.25 (1.06,1.41)] and stroke mortality [RR 1.58 (1.29,1.93)], stroke [RR 1.19 (1.02,1.40)], and type 2 diabetes incidence [RR 1.12 (1.03,1.21)] and cancers of the breast [RR 1.05 (1.01,1.10)] and esophagus [RR 1.46 (1.10, 1.95)].

We therefore summarized key GI/GL studies reported before and after the 2019 meta-analyses that formed the basis of the WHO report.

## Glycemic Index/Glycemic Load

Early studies demonstrated great differences in the GI of different foods, including the high GI of breads, whether white or whole grain flour, compared with spaghetti, either white or whole grain flour [1]. Similarly, legumes produced half the blood glucose rise compared with other carbohydrate-containing foods [1]. Low GI diets were shown to reduce hemoglobin A1c (HbA1c) over 6 mo compared with whole-grain wheat flour diets [7]. A similar picture was seen in a 3-y study with a reduction in HbA1c and body weight over the first 10 mo [8]. More recently a meta-analysis of 17 prospective cohorts demonstrated a significant increase in diabetes incidence related to a higher GI diet [RR 1.16 (95% CI: 1.06, 1.26)] [9]. Notably, the WHO review of GI and glycemic load (GL) [6] did not use the findings for GI and GL for energy adjusted cereal fiber in the large study by Bhupathiraju et al. [10] and instead used the unadjusted values.

A meta-analysis of randomized controlled trials (RCTs) that assessed the effect of low GI diets on multiple risk factors demonstrated significant reductions in HbA1c, plasma glucose, LDL cholesterol, BMI (kg/m^2^), and C-reactive protein (CRP) [11]. These findings may explain the earlier studies demonstrating the effect of high GL as an increased risk of diabetes [3] and coronary artery disease (CAD), especially among those with an elevated BMI [12].

Further cohort studies, for example, The European Prospective Investigation into Cancer and Nutrition (EPIC) cohort with 338,325 participants, demonstrated an association between GL and CAD especially in those with BMI > 25 (HR 1.22, 95% CI: 1.07, 1.40) [13]. Such associations have global relevance because a study using the international Prospective Urban and Rural Epidemiological study (PURE) cohort also demonstrated significant associations between GI and CVD and all-cause morality [14] and diabetes incidence [15]. In 2022, 2 other prospective cohort studies, the Shanghai men’s health studies (SMHS) and Shanghai women’s health studies (SWHS) combined (59,770 males and 74,735 females) found that higher GI and GL were associated with an increased risk of CVD mortality in Chinese adults [16]. The adverse effects of high GI for total mortality, CVD, and cancer were seen most clearly in females as reported in earlier studies. Additionally, in 2022, the meta-analysis by Dwivedi et al. [17] demonstrated associations, especially for GL and CAD.

Furthermore, a comprehensive review of meta-analyses by Miller et al. [18] implicated GL and 0GI as nutritional characteristics strongly linked to CVD and diabetes. Finally, in a recent meta-analysis of GI and GL data from large prospective cohort studies, >100,000 participants per cohort, GI/GL was related to diabetes and CVD and GI was also related to cancer incidence and all-cause mortality [19]. Of particular interest, although data were limited when the same very large cohorts were used for these comparisons, the magnitude of associations between GI, fiber, and whole grains and risks of diabetes, CVD, diabetes-related cancers, and all-cause mortality were not appreciably different (but in opposite directions).

These associations of GI/GL with diabetes incidence are further supported by trials with the alfa-glucosidase inhibitor acarbose. This drug slows the rate of carbohydrate digestion and absorption of glucose from starches and disaccharides [20], so producing a “physiologically low glycemic index diet.” Use of this drug in high-risk participants reduced the incidence of diabetes in the Study to Prevent Noninsulin-Dependent Diabetes Mellitus (STOP-NIDDM) and the Acarbose Cardiovascular Evaluation (ACE) randomized trials [21,22], indicating the importance of interventions that reduce daylong postprandial glycemic excursions even with no change in fiber or nutrient intake. These randomized trials provide strong evidence that the positive associations of GI and GL with diabetes are causal.

## Possible Mechanisms

Raised blood glucose even without diabetes is a risk marker for chronic disease [23]. One promising mechanism by which low GI diets may have multiple beneficial effects is through the reduction of oxidative stress metabolites [24]. Studies by Ceriello et al. [25] demonstrated reduced flow-mediated vasodilation and increased nitroprusside synthesis, as markers of increased oxidative stress, in clamp studies in which pulses of glucose were given. The changes in these markers of oxidative stress were inhibited by the infusion of vitamin C as an antioxidant. The importance of glycemic fluxes has been confirmed by continuous glucose monitoring in participants with diabetes whose daylong glucose fluctuations (mean ambulatory glucose excursions) over the day were found to be closely related to the urinary excretion of another marker of oxidative stress, 8-hydroxy prostaglandin F2 alfa [26]. These studies relate postprandial glycemic fluctuations to damaging reactive oxygen species that over time may be part of the reason for the elevated incidence of chronic diseases seen with high GI diets.

## Dietary Fiber and Whole Grains

The WHO report concludes that there was robust evidence for wide-ranging health benefits associated with higher intakes of fiber from cereals, vegetables, fruits, pulses, etc., and whole grains from prospective cohort studies, and RCTs (page 32 of the WHO report) [5].

There is a general agreement internationally that dietary fiber, and whole grains, have potential health benefits, but there is reason for concern over the terminology.

The term “fibers” should be used in the plural. This change in the name is important to promote the necessary broader thinking on this topic and is key for those considering search terms for meta-analyses. The plural form is important as fibers, such as in wheat bran, are largely insoluble and increase fecal bulk, whereas viscous fibers may reduce serum cholesterol but have little effect on fecal bulk. In other words, the different effects of the different types of fiber should be explored, and not all are classified under the simple term “dietary fiber.”

The term whole grain covers both the intact grain and the milled grain in the flour. This difference is important as the glycemic effects are very different for cooked intact grains (low GI) compared with the same grains as milled flour (high GI) in bread [27,28]. Thus, whole grain milled flour bread has a GI little different from white bread, although it may increase fecal bulking if eaten in sufficient quantity. Furthermore, increases in whole grain wheat flour in bread and breakfast cereal given to participants with diabetes over 3 months resulted in no change in HbA1c, indicating no effect on medium-term glycemic control of milled flour, whether as white or whole grain flour [29].

In conclusion, dietary fiber and whole grain-containing foods are likely to be protective for chronic disease outcomes, but more attention must be paid to nomenclature, and hence the classification of foods. The dietary GI and GL classifications relate directly and physiologically to specific foods, and also increased risk of chronic diseases, with higher GI/GL relating to increased risk. However, to better assess the health value of carbohydrate-rich foods, all 3 classifications should be used in combination.

## Author contributions

The authors’ responsibilities were as follows – DJAJ, WCW: responsible for the design, writing, and final content; and DJAJ, WCW: read and approved the final version.

## Funding

Banting and Best and the Karuna Foundation.

## Conflict of interest

DJAJ’s wife has previously been a director of INQUIS, which is responsible for clinical studies and GI testing. WCW declares that he has no conflict of interest to declare for this article.
